# A New Era in Cancer Care: How a Five-Minute Jab is Revolutionizing Treatment

**DOI:** 10.3126/nje.v15i3.88668

**Published:** 2025-12-31

**Authors:** Rajesh Elayedath, Fathima Naja

**Affiliations:** 1 School of Behavioural Sciences, Mahatma Gandhi University, Kerala, India; 2 Ex, Department of Clinical Pharmacy, MVR Cancer Centre and Research Institute, Kerala, India

Rapid and tangible changes in cancer treatment are uncommon. Now, throughout England, individuals who previously endured 30- to 60-minute IV sessions can finish their essential therapy within three to five minutes, skipping infusion equipment and lengthy waits for a swift injection before returning home [1]. Nivolumab, an established immunotherapy, is now offered as subcutaneous (SC) injection, making the NHS the first in Europe to provide this user-friendly option routinely for patients [1]. This initiative is not only a medical advancement but also marks a significant achievement for public healthcare by enhancing patient experience and boosting operational efficiency.

## From Breakthrough Molecule to Breakthrough Delivery

Nivolumab works by binding to and blocking the PD-1 protein, thereby preventing cancer cells from inhibiting the immune system, and activating the immune cells to attack the cancer; the drug is used to treat multiple types of cancer, such as melanoma, non-small cell lung cancer, bladder (urothelial) cancer, and esophageal cancer [2]. Hyaluronidase was added to the SC formulation to enhance its absorption, and it was thoroughly evaluated. The Phase 3 CheckMate-67T study established noninferiority in pharmacokinetics and objective response rate between SC nivolumab and IV in patients with advanced or metastatic clear cell renal cell carcinoma who had received prior treatment. The maintenance of health-related quality of life (HRQoL) over time in both arms was assessed by using such instruments as the FKSI-19 (total score LS mean difference of -0.5) and EQ-5D-5L VAS (LS mean difference of -0.2), proving this. This established equivalence is important. Clinicians would not consider abandoning the traditional IV technique and opting for an easier approach without being assured that treatment outcomes, and the HRQoL are maintained. The delivery mechanism can be developed with scientific certainty and become an integral part of everyday clinical practice.

## Patients at the Heart of the Change

The implications for patients are considerable. The results of the study demonstrate that patients preferred SC over IV injections by a remarkable margin (89.6 %), and cited reasons such as a feeling of independence, the ability to continue to live normally (50.7% of patients considered IV to be a burden to their families/caregivers, whereas only 24.9% considered this for SC), convenience of schedule, and emotional comfort (25.4% with SC, 50.2% with IV) [4]. Patient satisfaction was significantly greater with SC (78.6 percent satisfied to very satisfied) than with IV (33.3 percent), and patients were five times more likely to be bothered by the time of treatment with IV (36.8 percent very to extremely bothered) than with SC (7.0 percent). Also, fewer patients wished to switch from their assigned treatment with SC (23.9 percent) than with IV (40.8 percent) [4]. This is not merely a logistical change. It enables patients to have increased autonomy and dignity through treatment processes that are less invasive, and makes healthcare feel like a natural part of their lives. It shows that conquering cancer is not only possible but also can be integrated into daily life.

## An Efficiency Engine for the NHS

Patient comfort is not the only advantage, the NHS benefits in other ways, too. Approximately 1,200 individuals per month in England will benefit from this, saving more than 1,000 clinical hours per month, equivalent to the annual workload of multiple full-time staff members [1]. These newly freed-up hours allow healthcare professionals to shift their focus towards more crucial activities, increase unit capacity, and keep the costs unchanged because of the arrangements between NHS England and Bristol Myers Squibb [1]. This reflects value-based healthcare procurement, as a smarter application of biosimilars, and generics can be part of the NHS's value-from-medicines policy.

## A Strategic Step in a Larger Transformation

This advance facilitates the NHS's evolving model of decentralized, digitally empowered, and patient-centered care. The SC injection saves clinic time, which promotes a shift from hospitals to the community and allows for therapy to be conducted in more flexible environments. Redirected funds can enhance early detection and follow-ups, and reduce waiting lists, which are key objectives of the NHS Cancer Programme. This is similar to other developments, including AI-controlled robotic bronchoscopy for lung cancer diagnosis, AI-controlled MRI for the prostate, and community-based capsule sponge testing for the risk of oesophageal cancer [5].

## Why This Matters and What Comes Next

The implementation of SC nivolumab is an exemplary case of healthcare innovation, in which rigorous research, patient needs, and sustainable cost management are integrated. According to Professor Peter Johnson, the National Clinical Director of Cancer at NHS England, the impact of immunotherapy is viewed as a massive step forward in the sense that this technology makes things more convenient while saving thousands of valuable clinical hours every year [1]. This is not just efficiency; it represents a significant enhancement of patients' lives, and it does not imply any financial trade-offs. In the future, it is apparent that innovations that bring a new standard of care, faster access, and financial responsibility are crucial. As there is an increased prevalence of cancer, healthcare systems must accommodate the growing number of patients without placing extra pressure on resources. If the NHS adheres to evidence-based, patient-led, and cost-effective modifications, the five-minute injection can represent the initiation, not only of a new era in cancer care, but also of an era of flexibility, humanity, and sustainability in the public health sector.
